# Melanin Promotes Spore Production in the Rice Blast Fungus *Magnaporthe oryzae*

**DOI:** 10.3389/fmicb.2022.843838

**Published:** 2022-02-24

**Authors:** Pengyun Huang, Huijuan Cao, Yan Li, Siyi Zhu, Jing Wang, Qing Wang, Xiaohong Liu, Fu-Cheng Lin, Jianping Lu

**Affiliations:** ^1^State Key Laboratory for Managing Biotic and Chemical Threats to the Quality and Safety of Agro-Products, College of Life Sciences, Zhejiang University, Hangzhou, China; ^2^Institute of Plant Protection, Jiangsu Academy of Agricultural Sciences, Nanjing, China; ^3^Institute of Biotechnology, Zhejiang University, Hangzhou, China; ^4^State Key Laboratory for Managing Biotic and Chemical Threats to the Quality and Safety of Agro-Products, Institute of Plant Protection and Microbiology, Zhejiang Academy of Agricultural Sciences, Hangzhou, China

**Keywords:** *Magnaporthe oryzae*, melanin, sporulation, conidia, transcription factor, rice blast

## Abstract

The rice blast pathogen, *Magnaporthe oryzae*, spreads through spores and invades rice through appressoria. Melanin is necessary for an appressorium to penetrate plant cells, but there are many unknown aspects of its role in fungal conidiation. In this study, we confirmed that melanin promotes spore production in *M. oryzae*, and that this effect is related to the background melanin content of wild-type strains. In the wild-type 70-15 strain with low melanin content of aerial hyphae, increased melanin synthesis promoted sporulation. In contrast, increased melanin synthesis in the wild-type Guy11 strain, which has higher melanin content, did not promote sporulation. The transcription factor Cnf1 (conidial production negative regulatory factor 1), which negatively regulates melanin synthesis, has opposite effects in conidiophore differentiation of Guy11 and 70-15. Deletion of *CNF1* did not abolish the defects of Δ*cos1* and Δ*hox2* (where *COS1*/conidiophore stalk-less 1 or *HOX2*/homeodomain protein 2 was deleted) in conidiation, while increased the conidiation of Δ*gcc1* and Δ*gcf3* (where *GCC1*/growth, conidiation and cell wall regulatory factor 1, or *GCF3*/growth and conidiation regulatory factor 3 was deleted). Pig1 (pigment of *Magnaporthe* 1) regulates the melanin synthesis of hyphae but not of conidiophores, spores, or appressoria. Deletion of the same gene in different wild-type strains can lead to different phenotypes, partly because of differences in melanin content between fungal strains. Overall, this study reveals the functional diversity and complexity of melanin in different *M. oryzae* strains.

## Introduction

Rice blast, *Magnaporthe oryzae* (synonym *Pyricularia oryzae*), is a plant pathogen that seriously harms the production of rice, wheat, and other gramineous crops all over the world (Dean et al., 2012). It is also a model organism for studying plant-fungal interactions (Dean et al., 2012). Spore production and appressorium formation are two key steps allowing rice blast epidemics to spread. Rice blast spreads through spores that form appressoria on plant surfaces (Wilson and Talbot, 2009). The melanin layer is necessary for an appressorium to produce huge turgor pressure and penetrate the plant cuticle and cell wall in *M. oryzae* (Howard et al., 1991). Strains deficient in melanin synthesis are thus not pathogenic (Chumley and Valent, 1990). 1,8-dihydroxynaphthalene (DHN)-melanin is also a virulence factor for many pathogenic fungi, such as *Gaeumannomyces graminis*, *Colletotrichum lagenarium*, and *Pestalotiopsis fici* (Kubo et al., 1982; Money et al., 1998; Jacobson, 2000; Zhang et al., 2019). Some chemical fungicides, such as tricyclazole, pyroquilon, phthalide, and carpropamid have been used to prevent rice blast disease (Woloshuk et al., 1983). Tricyclazole is considered to function against rice blast disease by inhibiting melanin layer formation, although it also inhibits the sporulation of *M. oryzae* (Howard and Valent, 1996).

Melanin affects the morphology, cell wall structure, and stress resistance of fungal spores (Cordero and Casadevall, 2017; Zhu et al., 2021). However, the relationship between melanin and conidiation is complicated and elusive in fungi, especially *M. oryzae*. *Magnaporthe oryzae* has diverse field and laboratory strains with different colony colors and melanin contents. Melanin has diverse effects on sporulation in different *M. oryzae* strains. Deleting melanin synthesis genes has separate and individual effects on spore production in different strains (Zhu et al., 2021). For example, deleting melanin synthesis genes in *M. oryzae* strain Guy11 decreased fungal spore production, while deleting melanin synthesis genes in *M. oryzae* strain 70-15 had smaller effects on fungal conidiation (Zhu et al., 2021). The role of melanin in the sporulation processes among different fungi is also diverse. In the endophytic fungus *Pestalotiopsis microspore*, melanin negatively regulated conidia production, and deletion of *PKS1*, a polyketide synthase gene responsible for melanin synthesis, led to sixfold as many conidia as the wild type (Yu et al., 2015). In *P. fici*, overexpression of a melanin synthesis regulatory gene *PfMAH* increased spore production (Zhang et al., 2019). In the soilborne fungus *Verticillium dahlia*, however, deletion of *VdPKS1* or *VdCmr1*, a polyketide synthase or transcription factor (TF) gene responsible for melanin synthesis, did not affect fungal conidial development (Wang et al., 2018).

Many diverse genes are involved in the sporulation process in *M. oryzae*. For example, the Δ*hox2* mutant did not produce any spores, and 1,160 genes (8.5% of all genes in *M. oryzae*) were down-regulated or up-regulated more than twofold in Δ*hox2*, relative to the wild type (Kim and Lee, 2012). The Δ*cos1* mutant also did not produce any spores, and 442 genes (including melanin synthesis genes) were down-regulated or up-regulated more than twofold in Δ*cos1* (Zhou et al., 2009; Li et al., 2013). External environmental factors, such as nutrients, light, and moisture, affect fungal spore production by regulating the expression of many genes (Li and Kendrick, 1994; Kim and Lee, 2012). The numerous genes involved in the sporulation process have complicated analysis of the molecular mechanism of conidiation.

The TF Pig1 is required for mycelial melanin synthesis in *M. oryzae* (Tsuji et al., 2000). Deletion of *PIG1* in *M. oryzae* strain 70-15 did not significantly alter its spore production (Lu et al., 2014), while deletion of the TF *CNF1* in 70-15 led to more melanin content in the mutant’s mycelium and greatly increased conidiophore differentiation and spore production (Lu et al., 2014). We speculate that melanin is involved in promoting spore production, but it is not necessary for sporulation in *M. oryzae*. The 70-15 strain is a descendant of crosses between Guy11 and the progeny of rice isolate 104-3 and isolate AR4 from weeping love grass (Chao and Ellingboe, 1991). In this study, we demonstrated that Pig1 and Cnf1 have similar roles in hyphal melanin synthesis between two *M. oryzae* strains Guy11 and 70-15, but different roles in conidiation. In the *M. oryzae* strain 70-15 with lower melanin content, increased melanin content promotes fungal conidiation, while in strain Guy11, which always has high melanin content, continuing to increase melanin content no longer promotes conidiation.

## Materials and Methods

### Strains and Culture Conditions

The *M. oryzae* wild-type strains 70-15 (Chao and Ellingboe, 1991), Guy11 (Chao and Ellingboe, 1991), and 2539 (Leung et al., 1988), and all derivative strains were cultured on complete medium (CM) in a light incubator with an interval of 16 h light and 8 h dark at 25°C. Of the mutants, Δ*cnf1*_70–15_, Δ*rsy1*_70–15_, Δ*rsy1*_*Guy11*_, Δ*pig1*_70–15_, Δ*pig1*_*Guy11*_, Δ*gcf3*_70–15_, Δ*gcc1*_70–15_, Δ*cos1*_70–15_, and Δ*hox2*_70–15_ were generated in previous studies (Lu et al., 2014; Cao et al., 2016; Zhu et al., 2021). Cnf1, Pig1, Gcf3, Gcc1, Cos1, and Hox2 are transcription factors, and Rsy1 is a scytalone dehydratase in the fungal melanin synthesis (Lu et al., 2014; Cao et al., 2016; Zhu et al., 2021). To harvest aerial mycelium, conidia were collected from 7 to 12 day culture and washed once with distilled water. Then, 200 μl of a 5 × 10^4^ spores/ml spore suspension was spread on 9-cm CM plates and cultured for 5 day in a light incubator.

### Targeted Gene Knockout and Complementation

All targeted gene-deletion constructions were generated using a ClonExpress II One Step Cloning Kit (Vazyme Biotech, China) as described previously (Zhu et al., 2021). The 1-1.2 kb sections upstream and downstream of the targeted gene’s CDS region were amplified with a pair of primers (Supplementary Table 1) from the wild-type 70-15 genomic DNA. To obtain knockout vectors, upstream and downstream DNA fragments and the resistance gene were inserted into the *Xba*I-*Hin*dIII site of pKO3B containing a suicide gene *HSVtk* (Yan et al., 2019). Bialaphos resistance gene (*BAR*), Sulfonylurea resistance gene (*SUR*), hygromycin B resistance gene (*HPH*), and benomyl resistance gene (*BMLR*) were used in the knockout of *CNF1* in Guy11 or Δ*rsy1*_70–15_ (*BAR*), *PMK1* in 70-15 (*SUR*), *PIG1* in Δ*cnf1*_70–15_ or Δ*cnf1*_*Guy11*_ (*HPH*), and *GCC1*, *GCF3*, *COS1*, or *HOX2* in Δ*cnf1*_70–15_ (*BMLR*). The knockout vectors were transformed into *M. oryzae* through *Agrobacterium tumefaciens* mediated transformation (ATMT). The transformants were screened on the corresponding selective medium containing 0.5 μM 5-fluoro-2′-deoxyuridine (F2dU) and 150 μg/ml glufosinate ammonium, 100 mg/ml sulfonylurea, 200 mg/ml hygromycin B, or 10 μg/ml benomyl (Lu et al., 2014; Yan et al., 2019). The deletion of targeted gene in null mutants and the correct recombination of the resistance gene at the target gene locus were confirmed by PCR (Supplementary Figure 1) and the copy number of the resistance gene in null mutants was determined by quantitative PCR (qPCR) (Supplementary Table 2) as described previously (Lu et al., 2014; Cao et al., 2016). The phenotype of the mutants was confirmed by complementation.

For complementation of *RSY1* in mutants, the native *RSY1* gene including its promoter and terminator was amplified with primers listed in Supplementary Table 1 from 70-15 genomic DNA and inserted into the *Xba*I and *Hin*dIII linearized pKO1B-HPH (Lu et al., 2014). Transformants of Δ*cnf1*Δ*rsy1*_70–15_ complemented with *RSY1* were generated through ATMT, screened on CM plates containing 200 mg/ml hygromycin B, and confirmed by RT-PCR at the mRNA level.

### Growth, Conidiation, and Conidiophore and Appressorium Formation Assays

Phenotypic assays were performed according to previous reports (Lu et al., 2014; Cao et al., 2016). For the vegetative mycelium growth, colony morphology, and sporulation, 0.5-cm mycelial blocks were inoculated on CM plates and cultured for 8 days. At least five biological replicates were performed for each treatment. For appressorium formation, 25 μl conidia droplets (1 × 10^5^ spores/ml) were dropped on the artificial hydrophobic membrane and incubated at 22°C for 24 h under humid conditions. Three biological replicates were performed for each treatment with more than 200 conidia observed in each replicate.

The observation of conidiophores differentiated from aerial hyphae and conidia on a single conidiophore was performed with two methods as follows. (1) A thin slice of mycelial agar block was cut from the edge of a colony grown on CM agar, placed on a sterile glass slide, and incubated in a moisture chamber for 2–3 days under continuous light. After carefully removing the agar block, the aerial mycelium left on the glass slide was stained with 1–2 drops of lactophenol aniline blue (Hopebio, China), and then immediately observed and recorded with a light microscope (Nikon Eclipse Ni, Japan). The aerial hyphae were stained blue immediately, but conidiophores were not. (2) To observe the conidia formed on conidiophores, a few drops of spore suspension were inoculated on a sterile glass slide covered with a thin layer of CM agar covering on a sterile glass slide. The slides were kept in a moisture chamber for 3–4 days under continuous light. The conidiophores and their conidia were observed using a light microscope (Leica, Germany).

### Quantitative Analysis of Melanin Content

The melanin content assay was performed as described previously (Zhu et al., 2021). To develop a standard curve of melanin content, melanin was isolated from a culture of *M. oryzae* 2539 strain (it produces more melanin than 70-15 and Guy11). The fungus was cultured in PDB (potato dextrose broth) liquid medium for 12 day at 25°C, 150 rpm. To precipitate melanin, the same volume of 0.4 M HCl was added to the culture medium and further incubated at 4°C for 3 day. Then melanin was harvested through centrifugation at 10,000 *g* for 20 min, then successively washed twice with 0.1 M HCl and twice with distilled water and finally freeze-dried. To prepare azure A working solution, azure A (Sigma, United States) was dissolved into 0.2 M HCl, and diluted to an OD_610_ of 0.6. Gradient mass of melanin was added into 4 ml azure A working solution, and incubated for 30 min. The OD_610_ of each reaction was detected and recorded to establish the standard curve of OD_610_ and melanin content. Every concentration was repeated three times. To detect melanin in different strains, and exclude the soluble components in mycelium, aerial hyphae were sequentially ground into powder in liquid nitrogen, dissolved in water, centrifuged to remove supernatant and finally freeze-dried. About 2 mg hyphae powder was used to react with 4 ml azure A working solution for 30 min. Then melanin content in hyphae was calculated according to the OD_610_ value using the standard curve. Three biological replicates were performed in every strain.

### Phosphorylation Level Detection of Pmk1 and Mps1

Fungal proteins were extracted through combined trichloroacetic acid (TCA)/acetone precipitation, and a phenol extraction method as described previously (Wu et al., 2014). Aerial hyphae were disrupted in liquid nitrogen, suspended in 1 ml of precooled TCA/acetone (10% TCA in acetone) at −20°C overnight, and centrifuged at 10,000 rpm, 4°C, for 10 min to remove the supernatant. The protein pellet was washed with 0.1 M ammonium acetate in 80% methanol and then with 80% acetone, and dried for 20 min at room temperature. The protein pellet was resuspended in 500 μl SDS extraction buffer (2% SDS, 30% sucrose in 0.1 M Tris–HCl, pH 8.0), an equal volume of phenol equilibrated with Tris–HCl (pH 8.0) was added, and the sample was vortexed for 10 min then centrifuged at 10,000 rpm for 10 min at 4°C. Then the top phenol layer (0.3 ml) was transferred to a new tube, and 1.5 ml 0.1 M ammonium acetate in 80% methanol was added and incubated at −20°C overnight. The proteins were precipitated by centrifugation, washed with 80% acetone, and dried for 10 min at room temperature. The protein pellets were redissolved in 8 M urea containing 2% SDS. The phosphorylation level detection of Pmk1 and Mps1 was performed using western blot as described previously (Zhu et al., 2021). Phospho-p44/42 MAPK antibody (#4370, Cell Signaling Technology, United States), p44/42 MAPK (Erk1/2) antibody (#9102, Cell Signaling Technology, United States), anti-ERK1/2 MAPK antibody (C-9) (sc-514302, Santa Cruz Biotechnology, United States), and anti-GAPDH antibody (R1208-3, HuaBio, China) were used to detect phosphorylated Pmk1 and Mps1, unphosphorylated Mps1, unphosphorylated Pmk1, and GAPDH, respectively. The western blot pictures were taken using Image Lab (Version 5.2) on a ChemiDoc MP imaging system (Bio-Rad, China). The grayscale values of the bands were determined using the software ImageJ (1.511j8). The ratio of phosphorylated Pmk1/Pmk1 and phosphorylated Mps1/Mps1 was used to refer to the phosphorylation level of Pmk1 or Mps1 (Li et al., 2017).

### Gene Expression Level Analysis

Total RNA was extracted from aerial mycelia using TRIzol Reagent (Thermo Fisher Scientific, United States) and quantified by a NanoDrop 2000 spectrophotometer (Thermo Fisher Scientific, United States). cDNA was synthesized with the PrimeScript™ RT reagent Kit (Takara, Japan). Fifty ng/μl cDNA was used in qPCR to quantify the gene expression level with SYBR Premix Ex Taq (Takara, Japan) on a Mastercycler ep realplex (Eppendorf, Germany) in each sample. *H3* and β*-TUBULIN* genes were used as reference genes to normalize the expression level of target genes. The primers for qRT-PCR are listed in Supplementary Table 2. Five biological replicates were performed for each treatment.

### Statistical Analysis

Tukey’s HSD test in Data Processing System (DPS) was used for all experimental data in this study (Tang and Zhang, 2013). Values were displayed as means ± SD (standard deviation). Different letters in each column indicated statistically significant differences (*p* < 0.05).

## Results

### Pig1 Decreases and Cnf1 Increases Melanin Synthesis in Hyphae of *Magnaporthe oryzae* Strains

**FIGURE 1:**
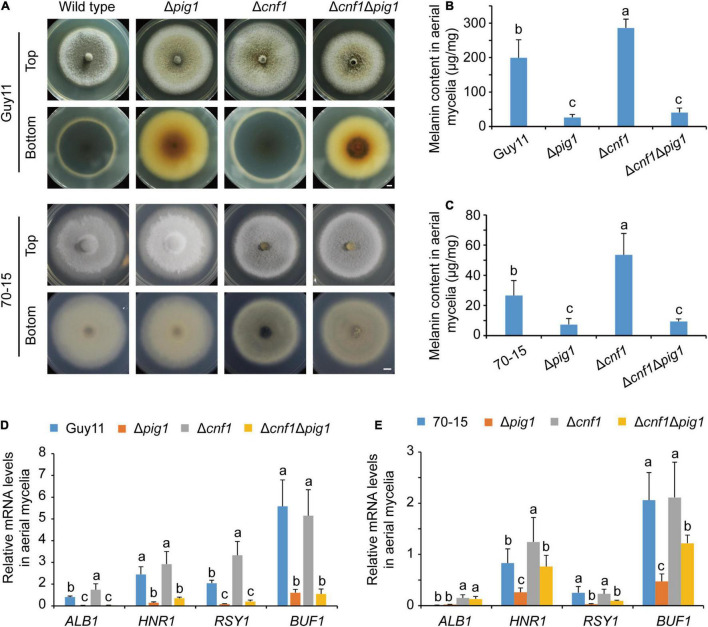
The different functions of Pig1 and Cnf1 on hyphal melanin synthesis in the rice blast fungus. **(A)** Colonial morphology and color. Bar = 5 mm. **(B)** Melanin contents in aerial mycelia of Guy11 and its mutants. **(C)** Melanin contents in aerial mycelia of 70-15 and its mutants. **(D,E)** Relative expression levels of four melanin synthesis genes (*ALB1*, *HNR1*, *RSY1*, and *BUF1*) in aerial mycelia of Guy11 **(D)** and 70-15 **(E)** with α-*ACTIN* and *40S* as reference genes. **(B–E)** Error bars represent ± SD. Different lowercase letters represent significant differences between four strains as estimated by Tukey’s HSD test (*p* < 0.05).

To characterize the diverse roles of Pig1 and Cnf1 in hyphae of the wild-type strains 70-15 and Guy11, we compared the phenotypes of the Δ*pig1*, Δ*cnf1*, and Δ*cnf1*Δ*pig1* mutants derived from 70-15 and Guy11. When cultured on CM, Δ*pig1*_Guy11_, Δ*cnf1*_Guy11_, and Δ*cnf1*Δ*pig1*_Guy11_ were brownish green on their spore-forming aerial mycelium sides, different from the black displayed by the wild-type Guy11. Δ*pig1*_Guy11_ and Δ*cnf1*Δ*pig1*_Guy11_ were yellow to brown on their substrate mycelium sides, while Guy11 and Δ*cnf1*_Guy11_ were black (Figure 1A). Δ*cnf1*_70–15_ and Δ*cnf1*Δ*pig1*_70–15_ were gray black, darker than that of the wild-type 70-15. However, Δ*pig1*_70–15_ was white on both sides of mycelia (Figure 1A). In addition, Δ*pig1*_Guy11_ and Δ*cnf1*_Guy11_ grew faster than Guy11, and Δ*cnf1*_70–15_ and Δ*cnf1*Δ*pig1*_70–15_ grew slower than 70-15 (Figure 1A).

We measured melanin contents in the spore-forming aerial mycelia of Δ*pig1*, Δ*cnf1*, and Δ*cnf1*Δ*pig1* derived from both wild-type strains Guy11 and 70-15 (Figures 1B,C). The spore suspensions were spread on cellophane films put onto CM, cultured for 5 days in an illumination incubator, and then mycelia were collected to extract melanin and RNA. In Guy11 lines, Δ*cnf1*_Guy11_ produced higher melanin content than the wild-type Guy11 significantly, and Δ*pig1*_Guy11_ and Δ*cnf1*Δ*pig1*_Guy11_ produced less melanin content than Guy11 significantly (Figure 1B). Similar to Guy11 lines, the melanin contents in aerial mycelia of Δ*cnf1*_70–15_ were higher than 70-15 significantly, and Δ*cnf1*Δ*pig1*_70–15_ and Δ*pig1*_70–15_ were lower than 70-15 significantly (Figure 1C). The melanin contents of aerial mycelia in Guy11 and its mutant lines were much higher than those in 70-15 and its mutant lines (Figures 1A–C). The melanin contents in Δ*cnf1*Δ*pig1* were similar to that in Δ*pig1* in corresponding wild-type strain (Figures 1B,C). The melanin content in mycelia of these two lines were consistent with the colony color, except Δ*cnf1*Δ*pig1*_70–15_. It showed a paradoxical appearance that the melanin content in the mycelia of Δ*cnf1*Δ*pig1*_70–15_ was significantly lower than Δ*cnf1*_70–15_, whereas these two strains displayed a similar colony morphology (Figures 1A,C).

We measured the expression levels of four melanin synthesis genes in the spore-forming aerial mycelia of Δ*pig1*, Δ*cnf1*, and Δ*cnf1*Δ*pig1* derived from wild-type strains Guy11 and 70-15. Relative to the wild-type Guy11, the expression levels of four melanin synthesis genes (*ALB1*, *HNR1*, *RSY1*, and *BUF1*) (Zhu et al., 2021) were significantly down-regulated in Δ*pig1*_Guy11_ and Δ*cnf1*Δ*pig1*_Guy11_ (Figure 1D). In Δ*cnf1*_Guy11_, the expression levels of *ALB1* and *RSY1* were significantly up-regulated; however, the expression levels of *HNR1* and *BUF1* were comparable to the wild-type Guy11 (Figure 1D). In 70-15 lines, the expression of *HNR1*, *RSY1*, and *BUF1* were significantly down-regulated in Δ*pig1*_70–15_ (Figure 1E). Compared with the wild-type 70-15, the expression levels of *ALB1* and *HNR1* were significantly up-regulated in Δ*cnf1*_70–15_, however, *ALB1* was up-regulated and *RSY1* and *BUF1* were significantly down-regulated in Δ*cnf1*Δ*pig1*_70–15_ (Figure 1E). Relative to Δ*pig1*_70–15_, the expression levels of *ALB1*, *HNR1*, and *BUF1* were significantly up-regulated in Δ*cnf1*Δ*pig1*_70–15_ (Figure 1E).

### Pig1 Does Not Control Melanin Synthesis in Spores and Appressoria

**FIGURE 2:**
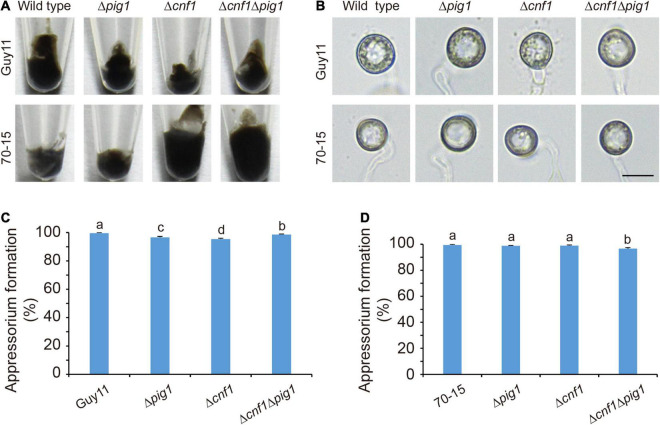
The functions of Pig1 and Cnf1 on melanin synthesis in spores and conidia. **(A)** Images showing spore color. **(B)** Images of appressorium morphology. Bar = 10 μm. **(C,D)** Appressorium formation rates in Δ*pig1*, Δc*nf1*Δ*pig1*, and Δ*cnf1* derived from the wild-type strains Guy11 and 70-15. **(C,D)** Error bars represent ± SD. Different lowercase letters represent significant differences between four strains as estimated by Tukey’s HSD test (*p* < 0.05).

Melanin is necessary for functional appressoria and virulence in *M. oryzae* (Howard and Ferrari, 1989; Howard et al., 1991), and Pig1 regulates melanin synthesis of the aerial mycelia in *M. oryzae* (Tsuji et al., 2000; Figure 1). However, Δ*pig1*_70–15_ still has full virulence in rice and barley (Lu et al., 2014). We speculated that Pig1 does not regulate melanin synthesis in appressoria, and then observed the melanin synthesis in spores and appressoria of Δ*pig1*, Δc*nf1*Δ*pig1*, and Δ*cnf1* derived from the wild-type strains Guy11 and 70-15. The spore colors of Δ*pig1*, Δc*nf1*Δ*pig1*, and Δ*cnf1* were similar to the wild type in both Guy11 and 70-15 derived mutants (Figure 2A). The color and morphology of appressoria formed by Δ*pig1*, Δc*nf1*Δ*pig1*, and Δ*cnf1* were comparable with their parent strains Guy11 and 70-15 (Figure 2B). Although some were statistically significant, the differences in appressorium formation rates between these mutants (Δ*pig1*, Δ*cnf1*Δ*pig1*, and Δ*cnf1* derived from the wild-type strains Guy11 and 70-15) and their wild type strains were small (Figures 2C,D).

### Pig1 and Cnf1 Have Different Roles in Conidiation in *Magnaporthe oryzae* Strains Guy11 and 70-15

**FIGURE 3:**
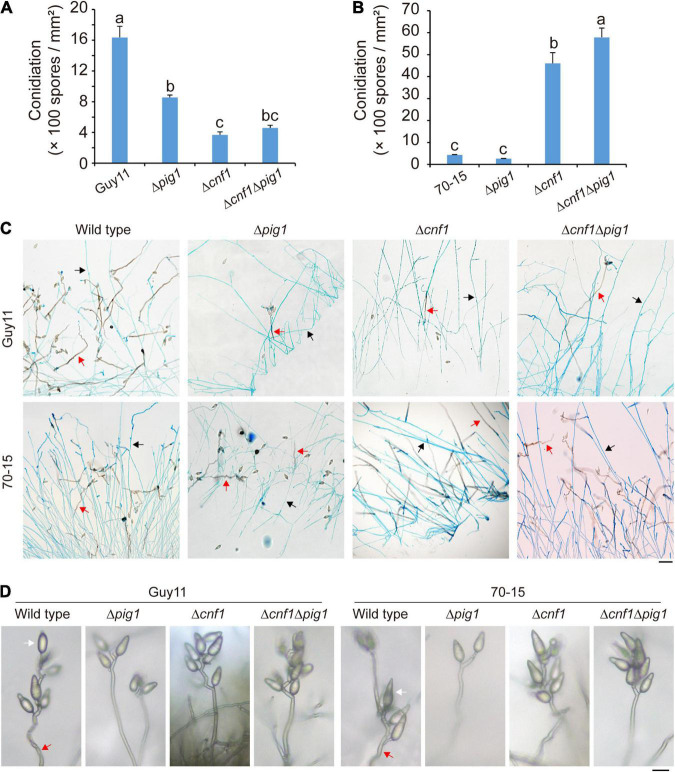
The different functions of Pig1 and Cnf1 on conidiation in *M. oryzae* strains Guy11 and 70-15. **(A)** Spore production of the wild-type Guy11 and its derived mutants (Δ*pig1*, Δc*nf1*Δ*pig1*, and Δ*cnf1*). **(B)** Spore production of the wild-type 70-15 and its derived mutants (Δ*pig1*, Δc*nf1*Δ*pig1*, and Δ*cnf1*). Error bars represent ± SD. Different lowercase letters represent significant differences between four strains as estimated by Tukey’s HSD test (*p* < 0.05). **(C)** Aerial hyphae and conidiophores formed by the wild-type Guy11 and 70-15, and their derived mutants (Δ*pig1*, Δc*nf1*Δ*pig1*, and Δ*cnf1*). The aerial hyphae were dyed blue by lactophenol aniline blue. Red arrows indicate conidiophores, and black arrows indicate aerial hyphae. Bar = 50 μm. **(D)** Conidiophores and conidia of the wild-type Guy11 and 70-15, and their derived mutants (Δ*pig1*, Δc*nf1*Δ*pig1*, and Δ*cnf1*). Red arrows indicate conidiophores, and white arrows indicate conidia. Bar = 20 μm.

In Guy11 lines, deletion of *CNF1* or *PIG1* or both in the wild-type Guy11 led to significantly decreased spore production in mutants (Figure 3A). In detail, the spore production of the wild-type Guy11 was 1.64 ± 0.15 × 10^3^ spores/mm^2^, and the spore production of Δ*pig1*_Guy11_, Δ*cnf1*_Guy11_, and Δ*cnf1*Δ*pig1*_Guy11_ were 0.86 ± 0.03 × 10^3^ spores/mm^2^, 0.37 ± 0.04 × 10^3^ spores/mm^2^, and 0.46 ± 0.03 × 10^3^ spores/mm^2^, respectively.

In 70-15 lines, different from Guy11 lines, deletion of *CNF1* in the wild-type 70-15 dramatically increased spore production, while deletion of *PIG1* did not affect spore production (Figure 3B). This result is consistent with our previous report (Lu et al., 2014). Deleting *CNF1* in Δ*pig1*_70–15_, Δc*nf1*Δ*pig1*_70–15_ produced more spores than the wild-type 70-15, Δ*pig1*_70–15_, and Δ*cnf1*_70–15_ (Figure 3B). In detail, the spore production of Δ*cnf1*_70–15_ and Δ*cnf1*Δ*pig1*_70–15_ after culturing on CM for 9 days were 4.60 × 10^3^ spores/mm^2^ and 5.79 × 10^3^ spores/mm^2^, respectively, about 10 and 13 times higher than that in the wild-type 70-15 (0.44 × 10^3^ spores/mm^2^) (Figure 3B).

The aerial hyphae and conidiophores were distinguished by staining with lactophenol aniline blue. The wild-type Guy11 and 70-15, and all of their mutants (Δ*pig1*, Δ*cnf1*, and Δ*cnf1*Δ*pig1*) formed conidiophores (Figure 3C). Guy11, Δ*cnf1*_70–15_, and Δc*nf1*Δ*pig1*_70–15_ differentiated more conidiophores than 70-15, Δ*cnf1*_Guy11_, and Δ*cnf1*Δ*pig1*_Guy11_ (Figure 3C). The conidiophores formed by Δ*pig1*_Guy11_, Δ*pig1*_70–15_, Δ*cnf1*Δ*pig1*_Guy11_, and Δc*nf1*Δ*pig1*_70–15_ were as dark as those of the wild-type Guy11 and 70-15 (Figures 3C,D). The number of conidia per conidiophore in Δ*cnf1* or Δc*nf1*Δ*pig1* was comparable with the wild-type strains (Figure 3D). Therefore, Cnf1 exclusively regulates conidiophore differentiation but is independent of conidium formation on conidiophores.

### Melanin Promotes Conidiation in *Magnaporthe oryzae* Strain 70-15

Deletion of *CNF1* in the wild-type 70-15 led to production of more spores and higher melanin content in aerial mycelia (Figures 1A,C, 3B; Lu et al., 2014), whereas Δ*cnf1*_Guy11_ produced fewer spores but had higher melanin content in aerial mycelia than the wild-type Guy11 (Figures 1B, 3A). 70-15 has much less melanin content than Guy11 in aerial mycelia (Figures 1B,C; Zhu et al., 2021). We proposed that the increase of melanin content in the Δ*cnf1*_70–15_ aerial mycelia promotes its spore production.

**FIGURE 4:**
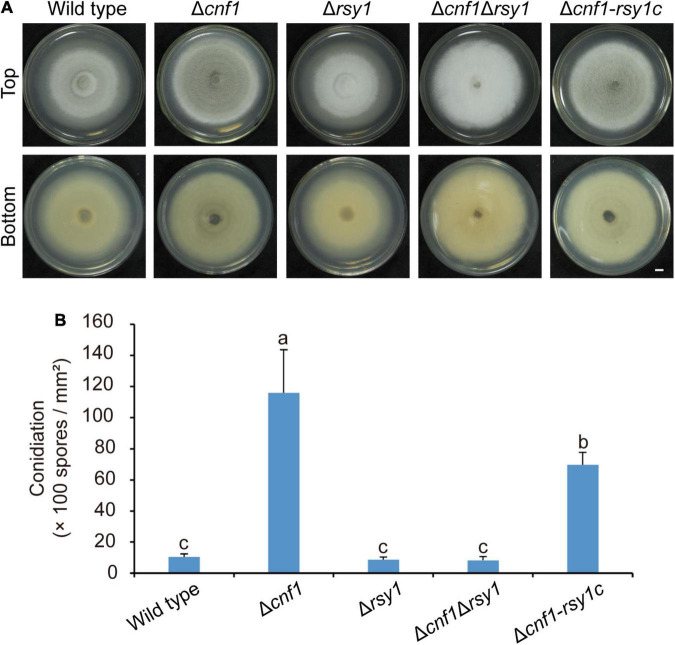
Melanin promotes conidiation in *M. oryzae*. **(A)** Images of colonial morphology and color of the wild-type 70-15 and its derived mutants (Δ*cnf1*, Δ*rsy1*, Δc*nf1*Δ*rsy1*, and Δ*cnf1-rsy1c*, a complementation strain of *RSY1* in Δc*nf1*Δ*rsy1*). Bar = 5 mm. **(B)** Spore production in the wild-type 70-15 and its derived mutants. Error bars represent ± SD. Different lowercase letters represent significant differences between four strains as estimated by Tukey’s HSD test (*p* < 0.05).

Rsy1 is a sole scytalone dehydratase in the melanin synthesis pathway in *M. oryzae* (Chumley and Valent, 1990). Deleting *RSY1* in the wild-type 70-15 resulted in loss of melanin, however, it did not alter conidiation in the mutant (Zhu et al., 2021). To test the role of melanin on fungal conidiation, we deleted *CNF1* in Δ*rsy1*_70–15_ to get Δ*cnf1*Δ*rsy1*_70–15_, and subsequently complemented Δ*cnf1*Δ*rsy1*_70–15_ with a native *RSY1* to obtain Δ*cnf1*-*rsy1c*_70–15_ (equivalent to Δ*cnf1*_70–15_). The mycelial color of Δ*cnf1*Δ*rsy1*_70–15_ was similar to Δ*rsy1*_70–15_, however, it was much whiter than the wild-type 70-15 and Δ*cnf1*_70–15_ (Figure 4A). Δ*cnf1*Δ*rsy1*_70–15_ produced as many spores as the wild type 70-15 or Δ*rsy1*_70–15_, but many fewer spores than Δ*cnf1*_70–15_ (Figure 4B). The complementation of *RSY1* in Δ*cnf1*Δ*rsy1*_70–15_ greatly increased melanin content in aerial hyphae (Figure 4A) and spore production (Figure 4B).

### Transcription Factors Gcc1 and Gcf3 Regulate Conidiation by Affecting Melanin Synthesis

**FIGURE 5:**
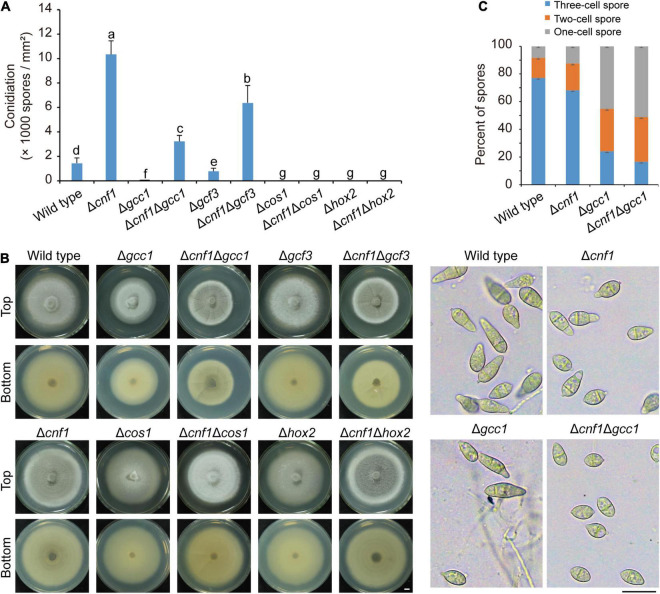
Diverse roles of five transcription factors in regulating conidiation and melanin synthesis in *M. oryzae*. **(A)** Conidiation in the wild-type 70-15, and its derived mutants (Δ*cnf1*, Δ*gcc1*, Δ*cnf1*Δ*gcc1*, Δ*gcf3*, Δ*cnf1*Δ*gcf3*, Δ*cos1*, Δ*cnf1*Δ*cos1*, Δ*hox2*, and Δ*cnf1*Δ*hox2*). Error bars represent ± SD. Different lowercase letters represent significant differences between strains as estimated by Tukey’s HSD test (*p* < 0.05). **(B)** Images of colonial morphology and color of the wild-type 70-15 and its derived mutants. Bar = 5 mm. **(C)** Spore morphology of the wild-type 70-15 and its derived mutants. Upper, percentage of spores with one cell, two cells, and three cells. More than 120 spores were counted for each strain. Error bars represent ± SD. Lower, spore morphology. Bar = 20 μm.

In previous reports, deletion of some TF genes, such as *COS1* (Zhou et al., 2009), *HOX2* (Kim et al., 2009; Liu et al., 2010), *GCC1*, and *GCF3* (Lu et al., 2014), led to defects in both conidiation and melanin synthesis. To find out how melanin synthesis affects conidiation, we deleted both *CNF1* and one of four TF genes (*COS1*, *HOX2*, *GCC1*, and *GCF3*) in the wild-type 70-15. Among these mutants, Δ*cnf1* produced more spores than the wild-type, Δ*cos1*_70–15_ and Δ*hox2*_70–15_ did not produce any spores (Figure 5A), and Δ*gcc1*_70–15_ and Δ*gcf3*_70–15_ produced fewer spores than the wild-type (Figure 5A; Lu et al., 2014). Deletion of both *GCC1* and *CNF1* or both *GCF3* and *CNF1* in the wild-type 70-15 led to significantly more spores than Δ*gcc1*_70–15_, Δ*gcf3*_70–15_ and the wild-type 70-15 (Figure 5A). However, Δ*cnf1*Δ*cos1*_70–15_ and Δ*cnf1*Δ*hox2*_70–15_ did not produce any spores as in Δ*cos1*_70–15_ and Δ*hox2*_70–15_ (Figure 5A). Colony colors of aerial mycelial in Δ*cnf1*Δ*gcc1*_70–15_, Δ*cnf1*Δ*gcf3*_70–15_, and Δ*cnf1*Δ*hox2*_70–15_ were blacker than 70-15, Δ*gcc1*_70–15_, Δ*gcf3*_70–15_, and Δ*hox2*_70–15_ (Figure 5B). Colonies of Δ*cnf1*Δ*cos1*_70–15_ produced more aerial hyphae than Δ*cos1*_70–15_ (Figure 5B). In addition, the size of spores of Δ*cnf1*Δ*gcf3*_70–15_ was smaller than that in 70-15. Most spores of Δ*cnf1*Δ*gcc1*_70–15_ and Δ*gcc1*_70–15_ were one- or two-celled while there were more three-celled spores in the wild type (Figure 5C).

### Deletion of *CNF1* Led to Activation of Pmk1 in *Magnaporthe oryzae* Strain 70-15

**FIGURE 6:**
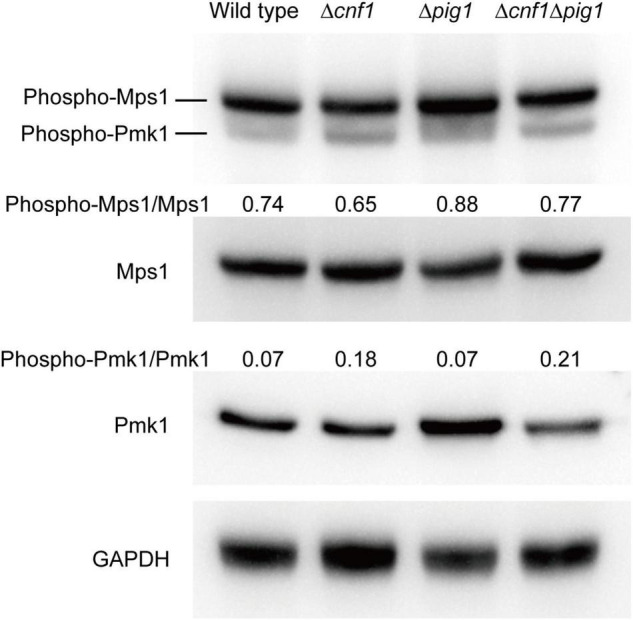
Analysis of phosphorylation level of Mps1 and Pmk1 in the wild-type 70-15 and its derived mutants (Δ*pig1*, Δ*cnf1*, and Δ*cnf1*Δ*pig1*) by western blot. Phosphorylated Pmk1 and Mps1 were detected using an anti-Phospho-p44/42 MAPK antibody. Total Pmk1 and Mps1 were detected using an anti-ERK1/ERK2 MAPK antibody. GAPDH was detected using an anti-GAPDH antibody. Numbers represent the protein contents of phospho-Pmk1 or phospho-Mps1 relative to total Pmk1 or Mps1.

**FIGURE 7:**
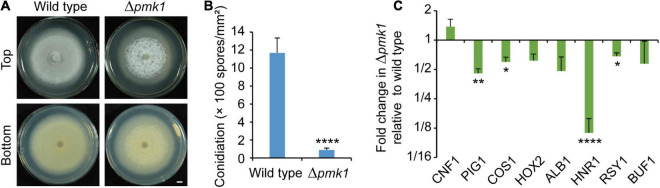
Pmk1 is required for regulation of melanin synthesis and conidiation. **(A)** Images of colonial morphology and color of the wild-type 70-15 and its derived mutant Δ*pmk1*_70–15_. Bar = 5 mm. **(B)** Conidiation in the wild-type 70-15 and its derived mutant Δ*pmk1*_70–15_. **(C)** Relative expression levels of two genes involved in conidiation (*COS1* and *HOX2*) and six melanin synthesis-related genes (*CNF1*, *PIG1*, *HNR1*, *ALB1*, *RSY1*, and *BUF1*) in aerial mycelia of Δ*pmk1*_70–15_. The data were shown after comparison with the wild-type 70–15. β*-TUBULIN* and *H3* were used as reference genes. **(B,C)** Error bars represent ± SD. Significant differences between the wild-type 70-15 and Δ*pmk1*_70–15_ were estimated by Tukey’s HSD test (*p < 0.05, **p < 0.01, and ****p < 0.0001).

Two mitogen-activated protein kinase (MAPK) genes, *MPS1* (*SLT2*) and *PMK1* (*FUS3*), were reported to regulate fungal melanin synthesis or conidiation in *Cochliobolus heterostrophus* (*PMK1*) and *Alternaria alternata* (*MPS1*) (Lev et al., 1999; Yago et al., 2011; Jiang et al., 2018). To see the effects on phosphorylation levels of Mps1 and Pmk1 by deleting *PIG1* and *CNF1*, we detected the phosphorylation levels in the aerial mycelia of Δ*pig1*_70–15_, Δ*cnf1*_70–15_, and Δ*cnf1*Δ*pig1*_70–15_ using western blot (Figure 6). The phosphorylation level of Mps1 was comparable among the four tested strains. The phosphorylation level of Pmk1 in the aerial mycelia of Δ*pig1*_70–15_ was comparable to that of the wild type. In Δ*cnf1*_70–15_ and Δ*cnf1*Δ*pig1*_70–15_, however, the phosphorylation level of Pmk1 was increased (Figure 6). To see the effects of Pmk1 on conidiation and melanin synthesis in *M. oryzae*, we measured the conidiation and mRNA expression levels of two genes involved in conidiation (*COS1* and *HOX2*) and six melanin synthesis-related genes (*CNF1*, *PIG1*, *HNR1*, *ALB1*, *RSY1*, and *BUF1*) in Δ*pmk1*_70–15_. Relative to the wild-type 70-15, the aerial hyphae in Δ*pmk1*_70–15_ were sparser and whiter (Figure 7A) and the spore production in Δ*pmk1*_70–15_ was significantly decreased (Figure 7B). *COS1*, *PIG1*, *HNR1*, and *RSY1* were significantly down-regulated in Δ*pmk1*_70–15_, relative to wild type (Figure 7C).

## Discussion

**FIGURE 8:**
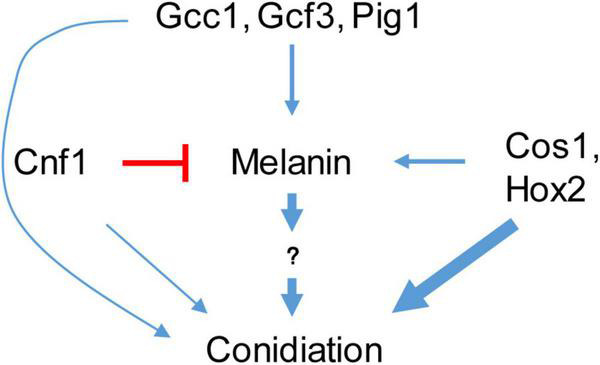
A schematic diagram of melanin and transcription factors regulating sporulation.

*Magnaporthe oryzae* spreads through spores and invades rice and other plants through appressoria (Wilson and Talbot, 2009; Dean et al., 2012). Melanin synthesis genes have diverse functions in the sporulation process of different *M. oryzae* strains (Zhu et al., 2021). Strains with the melanin synthesis gene deleted or mutated continue to produce spores, indicating that melanin synthesis is not necessary for rice blast sporulation (Chumley and Valent, 1990; Zhu et al., 2021). In some strains, deletion of melanin synthesis genes affects sporulation, while in other strains it does not (Zhu et al., 2021). Tricyclazole, an inhibitor of the melanin synthesis gene Buf1, inhibits both melanin synthesis and conidiation in *M. oryzae* (Yamaguchi et al., 1983; Kunova et al., 2013), indicating the relationship between melanin and conidiation. However, there is no clear conclusion about the relationship between melanin and sporulation (Chumley and Valent, 1990; Zhu et al., 2021). In this study, we confirmed that increasing the melanin content in *M. oryzae* strains with low melanin background content promotes their spore production (Figure 8).

In *M. oryzae* strain 70-15, deletion of the TF gene *CNF1* increased melanin synthesis, and also greatly increased spore production by differentiating more conidiophores but not spores on each conidiophore (Figure 3), indicating that Cnf1 is a regulatory factor inhibiting melanin synthesis and conidiophore differentiation in 70-15. This result is also supported by a previous report (Lu et al., 2014). In this study, we also knocked out *CNF1* in the wild-type strain Guy11. Melanin content in aerial mycelium of Δ*cnf1*_Guy11_ increased, but the spore production was significantly reduced compared with the wild-type Guy11. The mutant phenotypes of Δ*cnf1*_70–15_ and Δ*cnf1*_Guy11_ in melanin synthesis were similar, but the mutant phenotypes in spore production were opposite. In other words, the function of Cnf1 in regulating melanin synthesis was similar in Guy11 and 70-15, but the function in regulating spore production was contradictory between Guy11 and 70-15.

Δ*rsy1*_70–15_, a *RSY1*-deleted mutant in 70-15, could not synthesize melanin, but its spore production was not significantly different from that of the wild-type strain 70-15 (Zhu et al., 2021), indicating that the melanin synthesis gene *RSY1* is not necessary for spore production of *M. oryzae* strain 70-15. The melanin content in the mycelium of Δ*cnf1*_70–15_ is much higher than that of the wild-type strain, and the spore production is also greatly increased (Lu et al., 2014). After both *CNF1* and *RSY1* was deleted in the wild-type 70-15, the Δ*cnf1*Δ*rsy1*_70–15_ mutant could not synthesize melanin, but produced as many spores as Δ*rsy1*_70–15_ and the wild-type strain 70-15, which was much fewer than that of Δ*cnf1*_70–15_. When the *RSY1* gene was introduced into Δ*cnf1*Δ*rsy1*_70–15_, the spore production of the complementation strain (Δ*cnf1-rsy1c*) greatly increased. Therefore, melanin promotes sporulation of the 70-15 strain, but it is not sufficient or indispensable for sporulation.

The 70-15 strain is a laboratory strain derived from a series of genetic crosses (Chao and Ellingboe, 1991). A cross was made between strains 104-3 and AR4, and the progeny of them was then crossed/backcrossed with Guy11, a strain isolated from rice (Leung et al., 1988). The genomes of 70-15 and Guy11 have been sequenced separately (Dean et al., 2005; Bao et al., 2017). Bao et al. (2017) analyzed the sequence similarities and differences between the Guy11 genome and the 70-15 genome in detail. Most contigs of Guy11 were identical to the 70-15 genome and ran from end to end in a continuous main diagonal pattern (Bao et al., 2017). Although the genome of Guy11 is very similar with 70-15, there are still some differences between two strains. Many of the differences are related to the copy number and location of transposons. 108 large-scale structure variations were detected between the Guy11 and 70-15 genome, including 75 deletions, 12 insertions, 9 inversions, and 11 translocations (Bao et al., 2017). Phenotypically, Guy11 produces more melanin and more spores than 70-15 (Xue et al., 2012; Zhu et al., 2021). By deleting *CNF1* in the wild-type Guy11 to increase its melanin content, the Δ*cnf1*_*Guy11*_ mutant had not an increased spore production as in the 70-15 strain, but reduced fungal spore production. One of the reasons why the function of Cnf1 in regulating spore production is different in Guy11 and 70-15 may be that Guy11 and 70-15 differ in hyphal melanin contents.

Deletion of many TF genes reduces conidiation and results in whiter mycelium in *M. oryzae* (Kim et al., 2009; Lu et al., 2014; Cao et al., 2016). Δ*cos1* and Δ*hox2* did not produce any spores (Kim et al., 2009; Zhou et al., 2009; Liu et al., 2010), while Δ*gcc1* and Δ*gcf3* produced fewer spores (Lu et al., 2014). In 70-15 lines, deletion of both *CNF1* and *GCC1* or *GCF3* greatly increased mycelial melanin content and spore production. However, deletion of *CNF1* in Δ*cos1* or Δ*hox2* increased mycelial melanin content in Δ*hox2* and the number of aerial hyphae in Δ*cos1* but not spore production, suggesting that Cos1 and Hox2 are required for conidiation while Gcc1 and Gcf3 regulate fungal conidiation by affecting hyphal melanin content or number of aerial hyphae. In another *M. oryzae* strain, Y34, Cos1 regulated mycelial melanin synthesis but aerial hyphae did not differentiate into conidiophores (Zhou et al., 2009; Li et al., 2013). The homologs of Hox2 (Uvhox2 and Vhb1) are also involved in fungal asexual reproduction. In *Ustilaginoidea virens*, the *UvHOX2* mutant did not produce chlamydospores (Yu et al., 2019). In *Verticillium dahlia*, Δ*vhb1* produced fewer spores (Sarmiento-Villamil et al., 2018).

*CNF1* and *PIG1* double deletion mutants from 70-15 and Guy11 (Δ*cnf1*Δ*pig1*_70–15_ and Δ*cnf1*Δ*pig1*_Guy11_) showed different phenotypes in colony color and conidiation (Figures 1, 3). Moreover, the phenotypes of Δ*cnf1*Δ*pig1*_70–15_ displayed conflicts among sporulation, colony color, and melanin content (Figures 1–3). The aerial mycelial color is determined by both hyphal color and spore color. Δ*cnf1*Δ*pig1*_Guy11_ colonies are brown in color and it has lower mycelial melanin content. Δ*cnf1*Δ*pig1*_70–15_ has darker colonies but lower melanin content (Figure 1). Δ*cnf1*Δ*pig1*_Guy11_ produced much fewer spores than the wild-type Guy11 while Δ*cnf1*Δ*pig1*_70–15_ produced many more spores than the wild-type 70-15 (Figure 3). Therefore, the mycelial color of Δ*pig1*Δ*cnf1*_70–15_ (black) comes from the color of a large number of conidiophores and spores (black) rather than the hyphal color itself (white), however, the mycelial color of Δ*cnf1*Δ*pig1*_Guy11_ (brownish) mainly comes from the color of hyphae (brownish). Because the mycelial color of Δ*cnf1*_70–15_ comes from the color of both spores (black) and hyphae (black), although the mycelial color of Δ*pig1*Δ*cnf1*_70–15_ is similar to that of Δ*cnf1*_70–15_, its mycelial melanin content is much lower than that of Δ*cnf1*_70–15_ (Figure 1). One possible reason for these differences is that Pig1 is a key TF necessary for the regulation of melanin synthesis in hyphae but not in conidiophores and spores. There are no previous reports on the function of Pig1 or its homologs (Cmr1 and Amr1) in conidiophores. In *C. heterostrophus*, the orange-pink colony of Δ*cmr1* produced few spores, but its spores germinated and formed appressoria normally (Eliahu et al., 2007). In *A. brassicicola*, the colony of Δ*amr1* was orange and the virulence of spores was increased (Cho et al., 2012). In *C. lagenarium*, Δ*cmr1* formed reddish-brown colonies and spores germinated and formed melanized appressoria normally (Tsuji et al., 2000). In another *M. oryzae* strain 4091-5-8, the *pig1*^–^ mutant produced melanin in appressoria, but not in vegetative hyphae (Tsuji et al., 2000). In other studies of *M. oryzae* strain 70-15, the TF Pig1 also did not regulate the melanin synthesis of conidiophores, spores, or appressoria (Lu et al., 2014), while the TF Vrf1 specifically regulated the melanin synthesis of appressoria (Cao et al., 2016). No TFs that regulate the melanin synthesis of conidiophores and spores were identified in *M. oryzae* until the present study.

In summary, increased melanin synthesis promoted sporulation in *M. oryzae* strain 70-15, while in strain Guy11, increased melanin synthesis did not promote sporulation. In the 70-15 strain, the TFs Cnf1, Gcc1, and Gcf3 affected sporulation mainly by regulating the synthesis of melanin. However, the deletion of Cnf1 and Cos1 or Hox2 increased the synthesis of melanin, but didn’t promote the spore production of 70-15, indicating that Cos1 and Hox2 are key regulatory factors in the sporulation process that have priorities over the melanin content.

## Data Availability Statement

The original contributions presented in the study are included in the article/Supplementary Material, further inquiries can be directed to the corresponding author.

## Author Contributions

JL and PH contributed to experimental design. PH, HC, YL, SZ, JW, and QW contributed to experiments. PH, HC, SZ, and JL contributed to data analysis and scripts. F-CL, XL, and JL supplied experimental conditions. PH, JL, F-CL, and XL wrote the manuscript. All authors contributed to the article and approved the submitted version.

## Conflict of Interest

The authors declare that the research was conducted in the absence of any commercial or financial relationships that could be construed as a potential conflict of interest.

## Publisher’s Note

All claims expressed in this article are solely those of the authors and do not necessarily represent those of their affiliated organizations, or those of the publisher, the editors and the reviewers. Any product that may be evaluated in this article, or claim that may be made by its manufacturer, is not guaranteed or endorsed by the publisher.

## Supplementary Material

Supplementary Figure 1 Targeted deletion and complementation of genes in *M. oryzae* strains. **(A)** Strategy for targeted gene deletion. Two primer sets (upF/upR and dnF/dnR) were used to amplify two flanking fragments of targeted genes from the wild-type genome. The recombinant DNA fragment in null mutants was amplified by the LF/SeqR or SeqF/LR primer set. A partial fragment of the targeted gene in the wild type and transformants was amplified by the SF/SR primer set. **(B)** Knockout events were confirmed at the DNA level. The null mutants only showed a PCR band of ∼1,000 bp (representing β*-TUBULIN*, used as a positive control), while the wild type also had another band at about 300 bp (representing the targeted gene) (upper panel). Null mutants had a ∼2,000 bp-long recombinant DNA band, while the wild type did not (lower panel). **(C)** Complementation of the null mutants by their native genes corresponding to deleted genes. The complementation genes were confirmed at the mRNA level.
