# Supplementary figures and images for: Melanin Promotes Spore Production in the Rice Blast Fungus Magnaporthe oryzae
Source: Front Microbiol. 2022 Feb 24;13:843838. doi: 10.3389/fmicb.2022.843838 (PMC8920546; doi:10.3389/fmicb.2022.843838)

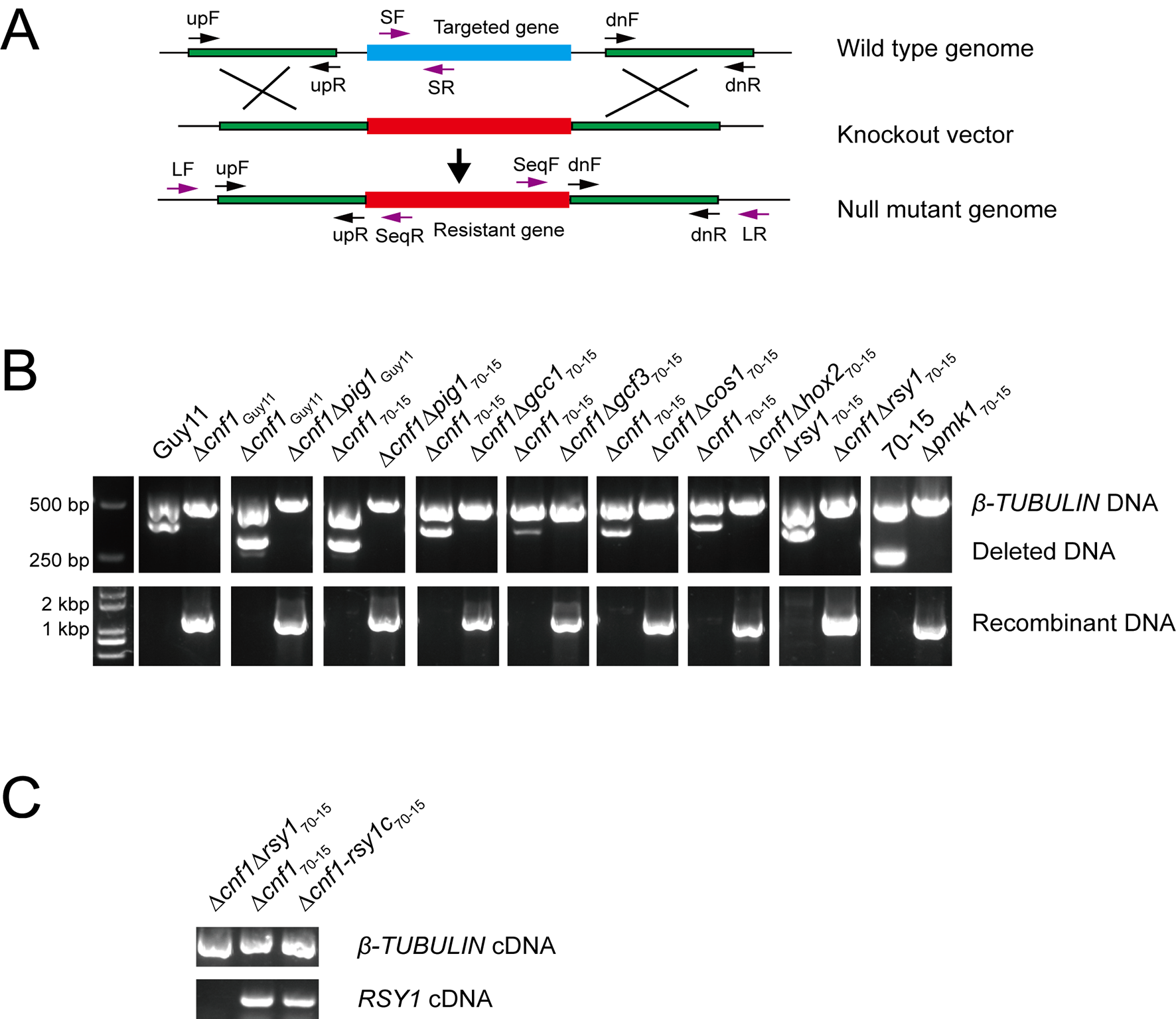

Supplement: Supplementary Figure 1 — Targeted deletion and complementation of genes in M. oryzae strains. (A) Strategy for targeted gene deletion. Two primer sets (upF/upR and dnF/dnR) were used to amplify two flanking fragments of targeted genes from the wild-type genome. The recombinant DNA fragment in null mutants was amplified by the LF/SeqR or SeqF/LR primer set. A partial fragment of the targeted gene in the wild type and transformants was amplified by the SF/SR primer set. (B) Knockout events were confirmed at the DNA level. The null mutants only showed a PCR band of ∼1,000 bp (representing β-TUBULIN, used as a positive control), while the wild type also had another band at about 300 bp (representing the targeted gene) (upper panel). Null mutants had a ∼2,000 bp-long recombinant DNA band, while the wild type did not (lower panel). (C) Complementation of the null mutants by their native genes corresponding to deleted genes. The complementation genes were confirmed at the mRNA level. [file Image_1.TIF]
