# Supplementary material for: Melanin Promotes Spore Production in the Rice Blast Fungus Magnaporthe oryzae
Source: Front Microbiol. 2022 Feb 24;13:843838. doi: 10.3389/fmicb.2022.843838 (PMC8920546; doi:10.3389/fmicb.2022.843838)
Supplement: Supplementary file 3 [file Table_2.DOCX]

**Supplementary Table S2. Copy identification of the resistant gene inserted in the mutant’s genome by qPCR.**

| Mutant | Copy numbers of a resistant gene^a^ | Insertion event |
| --- | --- | --- |
| Δ*cnf1*_Guy11_ | 0.93 | Single^c^ |
| Δ*cnf1*Δ*pig1*_Guy11_ | 1.03 | Single |
| Δ*cnf1*Δ*pig1*_70-15_ | 1.00 | Single |
| Δ*cnf1*Δ*gcc1*_70-15_ | 0.91 | Single |
| Δ*cnf1*Δ*gcf3*_70-15_ | 1.08 | Single |
| Δ*cnf1*Δ*cos1*_70-15_ | 1.06 | Single |
| Δ*cnf1*Δ*hox2*_70-15_ | 0.98 | Single |
| Δ*cnf1*Δ*rsy1*_70-15_ | 0.96 | Single |
| Δ*pmk1*_70-15_ | 1.01 | Single |

^a^ Copies of a resistant gene in the mutant genome were quantified by qPCR after normalization with the *β-TUBULIN* gene.

^c^ “Single” represents the targeted gene deletion event without ectopic insertion.
